# YOLOv9-Based Detection of Diseases in Poplar Trees Using Histogram Equalization and Computer Vision

**DOI:** 10.3390/s26113320

**Published:** 2026-05-23

**Authors:** Fazliddin Makhmudov, Kudratjon Zohirov, Jura Kuvandikov, Zavqiddin Temirov, Akmalbek Abdusalomov Bobomirzayevich, Mukhriddin Mukhiddinov, Khodisakhon Muraeva, Jasur Sevinov, Furkat Bolikulov

**Affiliations:** 1 Department of Computer Engineering, Gachon University, Sujeong-Gu, Seongnam-Si 461-701, Republic of Korea; furqat28@gachon.ac.kr; 2 Department of Software and Technical Support of Computer Systems, Karshi State Technical University, Karshi 180100, Uzbekistan; qzohirov@gmail.com; 3 Department of Computer Science and Programming, Jizzakh Branch of the National University of Uzbekistan Named After Mirzo Ulugbek, Jizzakh 130100, Uzbekistan; jorakuvandikov1@gmail.com; 4 Department of Digital Technologies, Alfraganus University, Yukori Karakamish Street 2a, Tashkent 100190, Uzbekistan; z.temirov@afu.uz; 5 Department of Artificial Intelligence, Tashkent University of Information Technologies Named After Muhammad Al-Khwarizmi, Tashkent 100200, Uzbekistan; bobomirzaevich@gmail.com (A.A.B.); mmuhriddinm@gmail.com (M.M.); h.murayeva@tuit.uz (K.M.); 6 Department of Artificial Intelligence, Tashkent State University of Economics, Tashkent 100066, Uzbekistan; 7 Department of Information Processing and Control Systems, Tashkent State Technical University, Tashkent 100095, Uzbekistan; j.sevinov@tdtu.uz; 8 Department of Robotics and Intelligent Systems, Tashkent University of Information Technologies Named After Muhammad Al-Khwarizmi, Tashkent 100200, Uzbekistan; 9 Department of Industrial Management and Digital Technologies, Nordic International University, Tashkent 100128, Uzbekistan; 10 Department of Computer Engineering, University of Tashkent for Applied Sciences, Tashkent 100149, Uzbekistan

**Keywords:** “poplar-disease” dataset, deep learning, histogram equalization, YOLOv9, object detection, Poplar (Populus) diseases

## Abstract

Poplar (Populus) trees are indispensable to various industries and environmental sustainability efforts. They are widely utilized for paper production, timber, and windbreaks, while also playing a significant role in carbon sequestration. Given their economic and ecological importance, the effective management of diseases is crucial. Convolutional Neural Networks (CNNs), renowned for their ability to process visual data, are pivotal in accurately detecting and classifying plant diseases. This study presents a domain-specific dataset of manually collected images of diseased poplar leaves from Uzbekistan and South Korea, ensuring geographic diversity and broader applicability. The dataset includes four disease classes, i.e., “*Parsha (Scab)*,” “*Brown spotting*,” “*White-Gray spotting*,” and “*Rust*,” which represent common afflictions in these regions. To advance research efforts, this dataset will be made publicly accessible, providing a valuable resource for the scientific community. Leveraging the cutting-edge YOLOv9c model, a state-of-the-art CNN architecture, we applied the Histogram Equalization technique as a preprocessing step to enhance the image quality to increase the accuracy of disease detection. This method not only improves the diagnostic performance of the model but also provides a scalable solution for monitoring and managing poplar diseases. By ensuring the health of poplar trees, this approach supports the sustainability of these critical resources. To our knowledge, this is the first publicly available dataset specifically focused on diseased poplar leaves, making it a significant contribution to global research efforts. It offers an invaluable resource for researchers and practitioners, enabling further advancements in early disease detection and sustainable forestry management.

## 1. Introduction

**Figure 1:**
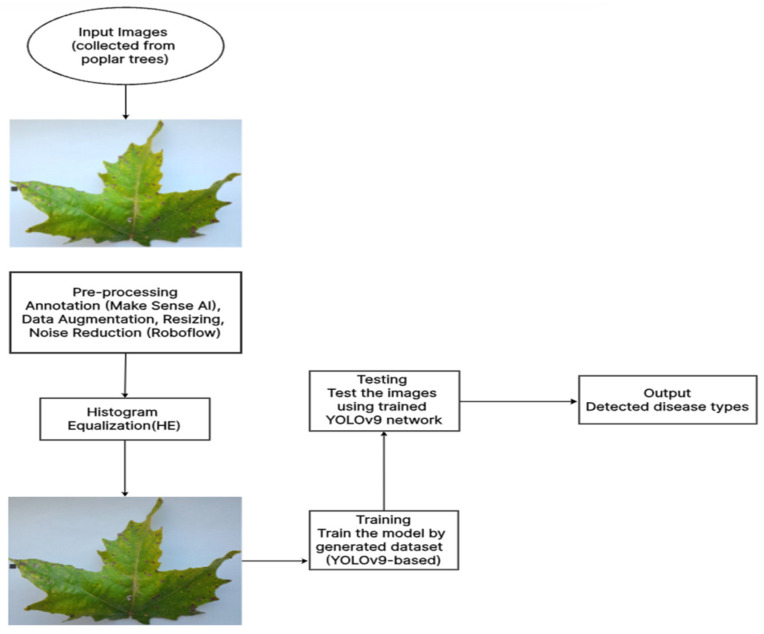
Overall workflow of the proposed poplar leaf disease detection framework.

Poplar (*Populus* spp.) is one of the most economically and ecologically important forest tree groups, with broad applications in timber production, biomass, environmental protection, and sustainable land management. In plant science, poplar is also regarded as a valuable model forest tree because of its rapid growth, ease of vegetative propagation, and relatively favorable genomic characteristics. As the role of poplar plantations expands in both industry and ecosystem restoration, maintaining tree health has become increasingly important for ensuring productivity, disease resilience, and long-term sustainability [1,2]. Among the main factors affecting poplar productivity are foliar diseases, which can reduce photosynthetic activity, weaken tree vigor, and ultimately lower economic yield. Early and accurate disease detection is therefore essential. However, traditional field inspection is time-consuming, subjective, and difficult to scale across large planting areas. For this reason, computer vision and deep learning methods are increasingly being adopted in plant disease analysis because they enable faster, more objective, and more automated diagnosis [3,4]. Recent studies related to poplar have demonstrated the growing potential of data-driven monitoring approaches. Hyperspectral analysis has been applied for non-destructive detection of poplar anthracnose [3], while multi-source time-series imaging combined with deep learning has been used for poplar seedling variety recognition and drought-stress classification [4]. In addition, recent work has shown the feasibility of applying modern object detection techniques such as YOLO-based models to poplar leaf disease analysis [5]. Beyond poplar-specific studies, related research on fruit-tree health monitoring has also confirmed the practical value of stereo imaging, UAV-based observation, and deep learning for disease detection under field conditions [6,7]. At the same time, the broader plant disease literature is evolving toward more lightweight, deployable, and intelligent systems. Recent work on CustomBottleneck-VGGNet has shown that compact CNN architectures can achieve high recognition performance while remaining suitable for deployment on resource-constrained devices [8]. SwinGhost-ClustNet further illustrates the value of combining transformer-based global-context modeling with lightweight convolutional feature extraction and explainable AI for plant disease recognition [9]. Similarly, LT-YOLOv10n demonstrates how lightweight object detection can be integrated with IoT infrastructure for real-time disease monitoring and management in smart agriculture [10]. In parallel, LeafDeSNet highlights the importance of improved feature selection and discriminative representation learning for multiclass plant leaf disease classification [11]. Despite these advances, research specifically focused on poplar leaf disease detection remains limited. Publicly available annotated datasets for diseased poplar leaves are still scarce, and relatively few studies have addressed lesion-level detection in this domain using modern deep learning pipelines. To address this gap, this study proposes a poplar leaf disease detection framework based on YOLOv9c and Histogram Equalization (HE). Histogram Equalization is used as a preprocessing step to improve contrast and make subtle lesion patterns more distinguishable, while YOLOv9c is employed as the detection backbone to identify disease regions efficiently. The proposed framework is trained and evaluated on a newly constructed annotated dataset containing four major disease classes: *Scab (Parsha), Brown Spotting, White-Gray Spotting*, and *Rust*. The overall workflow of the proposed framework is presented in Figure 1.

Our research advances poplar disease detection (Figure 1) by developing a YOLOv9c model trained on a newly curated dataset. While YOLOv9c and Histogram Equalization are individually well-established techniques, no prior work has applied their combination to poplar leaf disease detection. The contribution of this study is therefore primarily applied in nature: we demonstrate that this integration yields strong detection performance on a newly constructed, publicly available dataset, a resource that did not previously exist for this domain.

The primary contributions of this work are as follows: (1) We introduce the first publicly available annotated dataset for poplar leaf diseases, comprising images collected from three provinces in Uzbekistan and South Korea, covering four disease classes. (2) We demonstrate that Histogram Equalization, applied as a domain-specific preprocessing step, measurably improves detection accuracy for subtle lesion patterns in poplar leaves. (3) We establish a YOLOv9c-based detection pipeline optimized for this agricultural domain, providing a reproducible baseline for future research. The novelty of this study lies not in any single component but in its systematic integration and the resulting open dataset resource. The structure of this paper is carefully organized to ensure clarity and facilitate in-depth exploration of the topic as follows: Section 2 provides a comprehensive review of existing studies, highlighting the traditional methods used to identify key properties and indicators of poplar diseases. In Section 3, we introduce our proposed approach, detailing the methodology, the development of the YOLOv9c model, the application of histogram equalization (HE) for image enhancement, and the compilation of a robust popular disease dataset. Section 4 presents the experimental results obtained from testing our model with the newly created dataset, accompanied by a detailed analysis and discussion of the findings. Finally, Section 5 concludes the paper by summarizing the key contributions and outcomes of our work while proposing future directions to advance the field of poplar disease detection.

## 2. Related Works

In the digital technologies sphere for automated plant disease detection, prevailing methods fall into two broad categories: traditional Computer Vision techniques and more advanced approaches integrating Artificial Intelligence (AI), particularly Machine Learning (ML) and Deep Learning (DL). This discussion examines both. Crucially, system effectiveness depends not only on the chosen techniques but also on the availability of high-quality, well-structured datasets that enable accurate and efficient detection. In addition, integrating real-time processing and edge computing is an increasingly promising direction, improving scalability and responsiveness in agricultural settings.

### 2.1. Computer Vision and Image Processing Techniques for Detecting Diseases of Trees

A UAV-based pipeline for citrus tree detection using RGB imagery [12] demonstrates scalability for large orchards; however, it is limited to tree-level detection and does not address leaf-level disease classification, nor does it provide species-specific annotated datasets for forestry applications. A stereo-vision and deep learning framework for fruit tree leaf disease detection [6] achieves high mAP using binocular cameras and depth maps; however, the reliance on specialized stereo hardware increases system cost and reduces practicality for resource-limited field settings. A two-stage UAV system for apple tree health assessment [7] attains strong detection accuracy using multiband vegetation indices; however, its dependence on multispectral sensors makes it unsuitable for low-cost deployments, and the method is not transferable to poplar disease detection without significant retraining. A thermal imaging system for *apple scab* detection on embedded devices [13] demonstrates promising real-time performance; however, thermal cameras impose additional hardware requirements, and the approach has not been validated on foliar diseases of forest tree species such as poplar. A fluorescence spectroscopy method for HLB citrus disease detection [14] achieves high accuracy in controlled settings; however, it requires laboratory-grade imaging equipment and shows significant performance degradation across geographic domains (90% in Brazil vs. 61% in the USA), highlighting critical generalization limitations. A neutrosophic logic pipeline combined with Random Forest classification [15] achieves 98.4% accuracy for leaf disease identification; however, the method relies on handcrafted features and has not been evaluated on real-world field images or poplar-specific disease patterns. A computer vision-assisted GWAS study in *Populus* trichocarpa [3] applies semantic segmentation to quantify regenerating tissues; however, it focuses on plant biology rather than disease detection, and does not provide an annotated disease dataset applicable to the present task. Collectively, these studies demonstrate the potential of computer vision for plant health monitoring but share several critical limitations: dependence on costly or specialized imaging hardware (hyperspectral, multispectral, stereo, or thermal cameras), limited generalizability across tree species and geographic domains, and the absence of publicly available annotated datasets targeting poplar leaf diseases specifically. These gaps directly motivate our study, which proposes a practical and accessible approach based solely on standard RGB imagery enhanced with Histogram Equalization, and addresses the dataset gap by introducing the first publicly released annotated poplar leaf disease dataset covering four disease classes across two countries.

### 2.2. Machine Learning and Deep Learning Approaches for Tree Disease Detection

A comprehensive review covers ML and DL for plant disease detection, spanning NB, KNN, DT, SVM, RF, and MLP, as well as CNN-based architectures (e.g., Inception-v4, VGG-16). DL models consistently show superior performance for early, accurate diagnosis and yield protection [16]. G-RecConNN, a Gated-Recurrent Convolutional Neural Network, was introduced for early detection in plantain cultivation. CNNs extract spatial cues while GRUs model temporal progression across image sequences, enabling robust identification of diseases such as Black Sigatoka, Panama disease, and Bunchy top from real-time field datasets collected in Tamil Nadu, India [17].

Comparative studies show DL outperforming classic ML on citrus leaf disease classification: RF 76.8%, SGD 86.5%, SVM 87% versus DL models, VGG-19 87.4%, Inception-v3 89%, and VGG-16 89.5%—demonstrating DL’s advantage on complex image datasets [18]. Transfer learning for apple leaf diseases (*apple scab*, *cedar apple rust*, etc.) using fine-tuned VGG16, ResNetV2, InceptionV3, and MobileNetV2 achieved high accuracy, with ResNetV2 + Adam peaking at 94.7% despite class imbalance and environmental variability—underscoring practicality for precision agriculture [19]. A GoogLeNet-based CNN trained on PlantVillage achieved 98.54% overall accuracy for *Apple Scab*, *Apple Rust*, and *Apple Black Rot* (highest on *cedar rust*), illustrating the effectiveness of data augmentation and careful hyperparameter tuning [20]. In real-world walnut orchards, SSD detectors paired with ResNet50, Inception v2, and MobileNet v2 were used to identify anthracnose-infected leaves. ResNet50 delivered the best AP = 63%, demonstrating feasibility for real-time field detection while acknowledging remaining challenges under complex outdoor conditions [21,22,23,24,25,26,27,28].

## 3. Materials and Methods

### 3.1. Data Collection and Preprocessing

**Figure 2:**
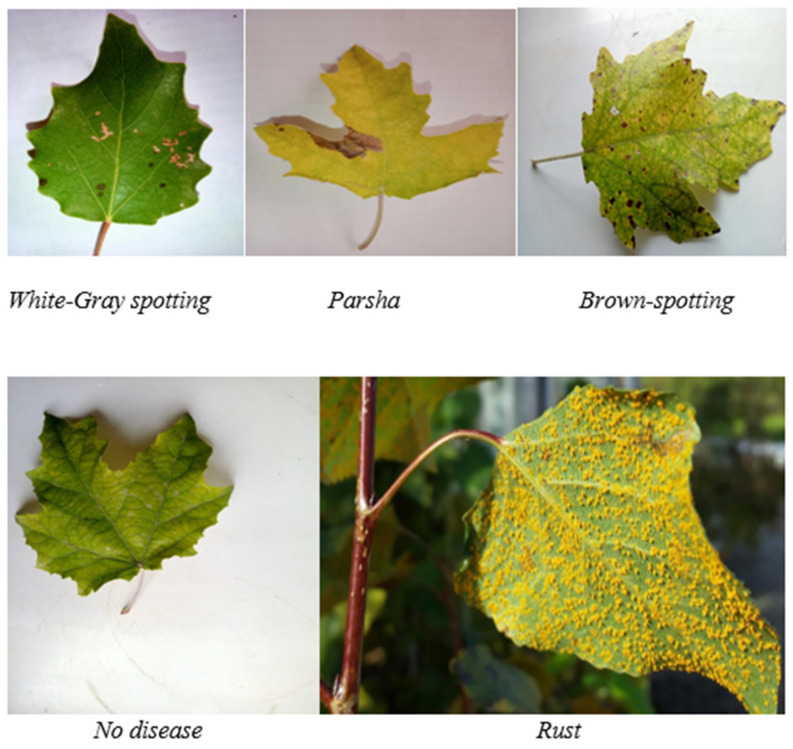
Disease types on the leaves of Poplar (Poplus).

One of the most critical factors in training an AI model is pairing a strong architecture with a high-quality dataset. Even with advanced models, insufficient data is a major bottleneck. We encountered exactly this challenge: no reliable, publicly available images of poplar leaves existed. Consequently, we created our own dataset, recognizing its value for future researchers. Considering environmental variability, we collected poplar leaves from three regions, Samarkand, Navoiy, and Jizzakh in Uzbekistan, and from South Korea during August and September, when leaves are fully mature and thus provide clearer disease cues. Guided by experts, we captured images of both healthy and diseased leaves using high-end cameras (12 MP sensors, f/1.6 aperture) to ensure sharp, detailed imagery even in low light. In total, we gathered 2357 images of poplar leaves. After 20 days of collection, our experts manually classified them into 362 healthy and 1995 diseased samples as illustrated in Table 1. The dataset targets four disease classes, *Scab* (*Parsha*), *Brown Spotting*, *White-Gray Spotting*, and *Rust*, which were selected in consultation with domain experts based on their frequency and visual distinctiveness in the collected images. With expert support, we then meticulously annotated diseased leaves for four target classes: * Scab*, *Brown Spotting*, *White-Gray Spotting*, and *Rust* as illustrated in Figure 2.

We utilized Make Sense AI [29] with a polygon-based structure to label diseased areas precisely. Notably, some leaves displayed multiple diseases, each annotated individually. These annotations were saved as JSON files, capturing the coordinates of each disease within the image. We then exported our labeled dataset in JSON format from Make Sense AI and proceeded to enhance it using Roboflow [30] through augmentation techniques. This included rotating images 90 degrees in both directions and minor rotations between −15 and +15 degrees [31,32], using Computer Vision techniques. The augmentation pipeline was limited to geometric transformations only, specifically rotations of ±15° and 90°, applied via Roboflow after the train/validation/test split. This decision was made to avoid introducing artificial color distortions, as the natural coloration of diseased regions is a critical diagnostic feature for accurate disease identification. It is important to note that the augmentation techniques were deliberately limited to geometric transformations (rotations of ±15° and 90°) to avoid introducing artificial color distortions, as the natural coloration of diseased regions is a critical diagnostic feature. Although the current dataset comprises 2357 original images, the geographic diversity spanning three provinces in Uzbekistan and South Korea introduces meaningful variation in lighting conditions, leaf morphology, and disease appearance. Nevertheless, we acknowledge that a larger and more diverse dataset would further improve the generalization capability of the model, and expanding the dataset across additional regions and seasons is planned as part of future work. Augmentation was applied exclusively to the training subset using Roboflow’s pipeline. The validation and test subsets were kept in their original, non-augmented form to ensure unbiased evaluation. As a result, the training set expanded to 4927 images (4057 diseased and 870 healthy), while validation and test sets retained their original sizes of 236 images each (200 diseased and 36 healthy). The total dataset size is 5399 images (Table 2) and was converted to a txt format compatible with YOLO, which requires txt-formatted data for training.

**Figure 3:**
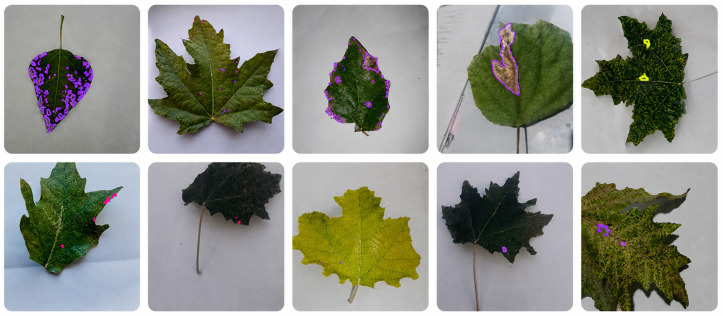
Samples of annotated poplar leaves prior to augmentation.

**Figure 4:**
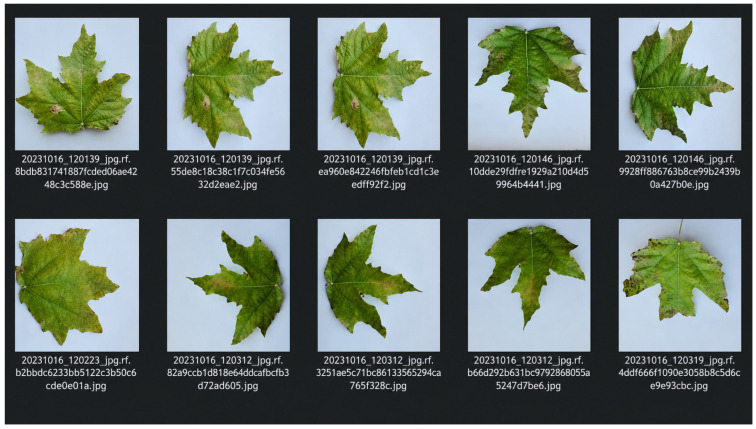
Sample images from the training dataset after the augmentation process.

This step was essential to prevent any color distortions, as preserving the natural color of the diseased areas on the leaves is critical for accurate disease identification. Following the application of these techniques, the total dataset size reached 5399 images. As illustrated in Figure 3 and Figure 4, all images underwent manual labeling, augmentation, and a significant increase in quantity to strengthen the machine’s learning potential. By maintaining the visual integrity of the diseased regions, this process ensures the model can detect diseases with higher precision.

### 3.2. Proposed Method and Model Architecture

Our primary aim is to improve disease detection in poplar leaves by leveraging an advanced detection model optimized with YOLOv9c. Plant disease detection is challenging because diseased regions are small, subtle, and often blend into surrounding textures due to diverse shapes and sizes, which reduces clarity and contrast. To counter this, we apply Histogram Equalization, a contrast-enhancement technique that redistributes intensity values to make lesions more prominent and improve detection accuracy [33]. Object detectors depend on clear, distinctive features, so variability in leaf appearance and symptoms can hinder traditional models. Moreover, real-time processing is essential for continuous data streams (e.g., live video), where latency can delay intervention. YOLOv9c is selected for its ability to handle diverse image aspect ratios while providing rapid, precise detection, addressing limitations of conventional approaches and improving both speed and reliability for real-time poplar disease identification.

### 3.3. The Model Structure of YOLOv9 Network

YOLOv9 is a powerful member of the YOLO family, optimized for high-speed, accurate detection in complex, real-time scenarios such as plant disease monitoring. Its enhanced backbone strengthens fine-grained feature extraction, while a Path Aggregation Network (PANet) fuses multi-level features to improve small-object detection. Integrated attention mechanisms focus the model on salient regions, boosting sensitivity to subtle disease spots. An anchor-free detection head enables direct prediction, improving efficiency and adaptability to objects with diverse shapes and sizes. Multi-scale prediction further aids detection across varying lesion dimensions. Finally, YOLOv9 is tuned for real-time operation, ensuring timely inference on continuous data streams, making it well suited for dynamic agricultural environments that demand rapid, precise detection.

**Figure 5:**
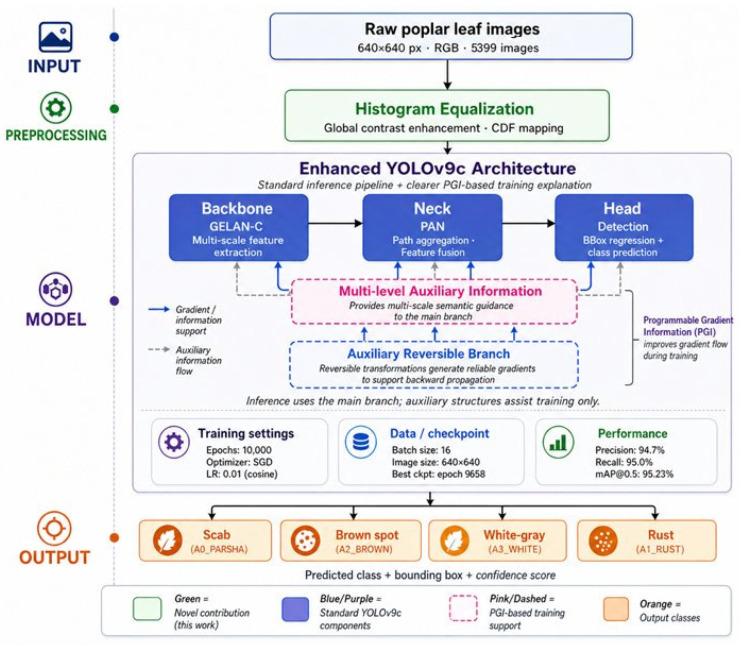
Detailed architecture of the proposed YOLOv9c-based detection model [35].

Figure 5 illustrates the YOLOv9c architecture used in this study for poplar leaf disease detection. The framework comprises a GELAN-C Backbone for extracting deep hierarchical and multi-scale image features, a PAN-based Neck for aggregating and fusing features from different resolution levels, and an Anchor-Free Detection Head for performing multi-scale bounding-box regression and category prediction. To further enhance training effectiveness, the architecture includes an Auxiliary Reversible Branch, which provides more stable and reliable gradient flow, and a Programmable Gradient Information (PGI) mechanism, which controls multi-level semantic guidance during training to improve feature learning and optimization. These modules collectively contribute to stronger representation learning and more accurate detection performance. The figure was adapted and redrawn from Wang et al. [34].

### 3.4. Techniques for Enhancing Image Quality

Improving image quality is essential in fields like photography, computer vision, and digital imaging, where techniques to boost contrast, sharpness, and visual clarity enhance the usability and esthetics of images. Here are some effective methods:

Histogram Equalization: This method redistributes intensity values to flatten the histogram, enhancing global contrast. It is particularly useful for images with uniformly bright or dark areas, making subtle details more visible in challenging lighting.

Contrast Stretching: This technique expands the intensity range of an image, transforming pixel values to cover the full 0–255 range in an 8-bit grayscale image. By adjusting the minimum and maximum values, it improves feature visibility, making details more discernible.

Adaptive Histogram Equalization (CLAHE): Unlike standard equalization, CLAHE operates on localized regions, or “tiles,” to enhance local contrast. This method is effective in images with varying light conditions, improving edge definition and detail in each area.

Gamma Correction: By applying a power-law transformation, gamma correction adjusts image brightness, enhancing both dark and bright areas. Selecting an appropriate gamma value significantly boosts visibility across different intensity ranges, fine-tuning brightness and contrast.

Unsharp Masking: This edge-enhancement technique sharpens an image by subtracting a blurred version from the original, emphasizing edges while maintaining smoothness. It is commonly used to improve image clarity in various digital imaging applications. These methods provide diverse solutions for improving image quality across various use cases, offering tailored approaches for images in need of global or localized enhancement, sharpness, and optimal brightness adjustments [36].

### 3.5. Histogram Equalization Technique

Histogram Equalization (HE) is a method used to improve the contrast in an image by spreading out the intensity values across the entire range. This technique redistributes the pixel intensities, making darker areas lighter and brighter areas darker, thus enhancing overall visibility. Here is how it works, along with the key formulas used in the process.

#### Step-by-Step Process of Histogram Equalization

Histogram Calculation: First, the histogram of the image is computed. This histogram counts the frequency h(i) of each intensity level i, where i ranges from 0 to L−1 (for an 8-bit image, L=256). This histogram represents the original distribution of pixel intensities across the image:

(1) h(i)=f(i)

where f(i) is the count of pixels at intensity i.

Probability Distribution: Next, the probability distribution p(i) for each intensity level is calculated by dividing the frequency f(i) of each intensity by the total number of pixels N in the image:

(2) $$p\left(i\right)=\frac{f(i)}{N}$$

This probability distribution provides a normalized measure of the intensity distribution within the image.

Cumulative Distribution Function (CDF): The CDF, C(i), is then calculated for each intensity level. The CDF is the sum of all probabilities from intensity 0 up to i, and it essentially represents a cumulative probability for each intensity level:

(3) $$C\left(i\right)=\sum_{j=0}^{i}p\left(j\right)$$

The CDF is crucial as it helps map the old intensity values to new, more balanced ones.

Intensity Mapping Function: Each intensity level i is then mapped to a new level $i^{\prime }$ using the cumulative distribution function, effectively spreading out the histogram across the available intensity range. This new level $i^{\prime }$ is calculated as:

(4) $$i^{\prime }=round(C\left(i\right)\times (L-1))$$

where L−1 is the maximum intensity level (255 for an 8−bit image). This mapping assigns new intensity levels that make the histogram more uniformly distributed.

Final Transformation: Finally, each pixel’s original intensity is replaced with the new intensity $i^{\prime }$ calculated from the mapping function, producing a new image with enhanced contrast. This step completes the transformation, resulting in a visually improved image with more distinguishable features and balanced brightness levels [37].

Enhanced Detail Visibility: By spreading out the intensity values, histogram equalization makes subtle discolorations and small lesions more visible. This can help identify diseases at an early stage, allowing for timely intervention. Improved Classification Accuracy: When used with machine learning or computer vision techniques, histogram-equalized images can improve the accuracy of automated disease classification by enhancing the contrast of key features.

**Figure 6:**
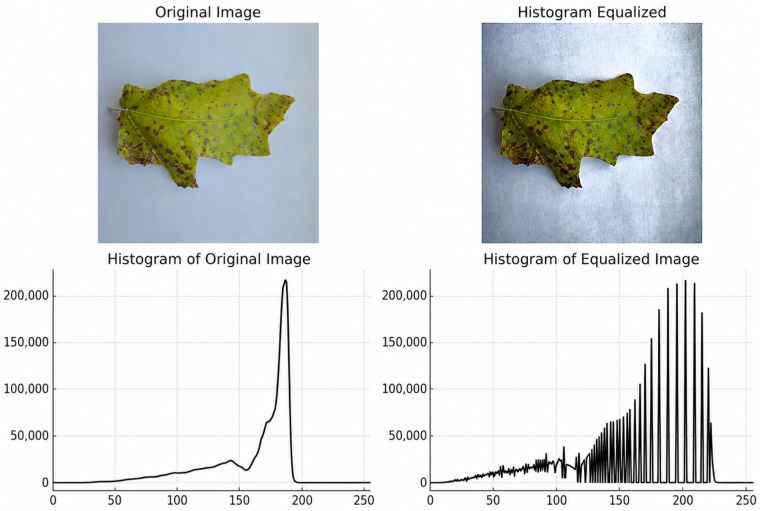
Histogram equalization effect on a poplar *diseased* leaf image.

Ease of Visual Inspection: For human inspectors, histogram equalization simplifies the task of distinguishing between healthy and infected tissues, aiding in manual assessment of disease severity. The image darkening was increased by 50% through the adjustment of histogram equalization parameters showed on the Figure 6. This technique not only improves the visual quality of images for human inspection but also provides a more robust dataset for training automated disease-detection systems [38].

### 3.6. Implementation Details

All experiments were implemented in Python 3.9.12 using PyTorch 2.2.1 with CUDA 11.8. Training was performed on an NVIDIA RTX 4060 Ti (16 GB). The YOLOv9c model was trained from scratch with the following hyperparameters: input image size 640 × 640, batch size 16, SGD optimizer, initial learning rate 0.01, momentum 0.937, weight decay 0.0005, and cosine learning rate annealing over 10,000 epochs. The extended training schedule was chosen because the model was trained from scratch without pretrained weights, requiring more epochs to converge than fine-tuning-based approaches. Training logs confirm that the model continued to improve beyond epoch 8000 (mAP@0.5: 0.9475), reaching its best validation mAP@0.5 of 0.9523 at epoch 9658. The cosine annealing schedule was designed to span the full training duration, and the best checkpoint strategy ensured that the globally optimal model was selected. No early stopping was applied; the checkpoint with the highest validation mAP@0.5 was selected for final evaluation. All code and the dataset will be made publicly available upon acceptance.

## 4. Experimental Results

This study lays a strong foundation for effective detection of diseases in poplar (*Populus*) leaves, demonstrating the significant potential of Computer Vision and Deep Learning (DL) to address critical agricultural health challenges. By rigorously evaluating performance with Precision, Recall, and F1 score, we gain a comprehensive view of accuracy and robustness, supporting real-world applicability for poplar disease management. The promising initial results motivate continued research and refinement to further improve accuracy and scalability, enabling broader agricultural deployment.

### 4.1. Model Evaluation

We evaluate the model using the confusion matrix, a fundamental tool that compares predictions against ground truth to characterize detection accuracy. This provides clear insight into the model’s precision in distinguishing diseased from healthy leaves. Metric selection is tailored to data properties and research objectives, ensuring a balanced assessment of effectiveness. We focus on the core counts, True Positives (TP), True Negatives (TN), False Positives (FP), and False Negatives (FN), as they capture the model’s ability to correctly classify leaf conditions. These indicators, along with derived measures such as Precision, Recall, and F1 score, reveal strengths and limitations and guide targeted improvements to enhance detection accuracy [39].

(5) $$Precision=\frac{TP}{\left(TP+FP\right)}$$

(6) $$Recall=\frac{TP}{\left(TP+FN\right)}$$

(7) $$F1\;score=\frac{\left(2\times Recall\times Precision\right)}{\left(Recall+Precision\right)}$$

Assessing these metrics enables a comprehensive evaluation of the model’s effectiveness, underscoring its usefulness and reliability across deployment contexts. Analyzing these indicators reveals both capabilities and limitations, which is essential for determining practical suitability and ensuring accurate, consistent detection of diseases on poplar leaves. In this study, testing accuracy is defined as image-level classification accuracy, the percentage of test images in which at least one disease region was correctly detected and classified.

### 4.2. Experimental Results and Discussion

In object detection, the YOLO (You Only Look Once) architecture is widely used for identifying both dynamic and static objects. Its applications span license plate recognition, pedestrian and wildlife detection, and leaf analysis to localize affected regions and assess plant health. This versatility stems from YOLO’s use of deep convolutional neural networks (DCNNs) to efficiently learn and detect discriminative features. To address disease detection in poplar (*Populus*), we trained on a newly developed dataset comprising 5399 images in total, of which 4927 are training images (4057 diseased and 870 healthy) and 472 are held-out validation and test images (400 diseased and 72 healthy) kept in their original, non-augmented form. All experiments were implemented in Python 3.9.12 using PyTorch 2.2.1 with CUDA 11.8 on the workstation described in Table 3. The YOLOv9c model was trained from scratch with the following hyperparameters: input image size 640 × 640, batch size 16, SGD optimizer, initial learning rate 0.01, momentum 0.937, weight decay 0.0005, and cosine learning rate annealing over 10,000 epochs. No early stopping was applied; the checkpoint with the highest validation mAP@0.5 was selected for final evaluation. All code and the dataset will be made publicly available upon acceptance [40].

Leveraging advanced deep learning framework such as PyTorch, we developed and trained a robust detection model tailored to identify diseases in poplar (*Populus*) leaves. After a comprehensive training regimen, we rigorously evaluated performance using accuracy, precision, recall, and F1 score to provide an overall assessment.

**Figure 7:**
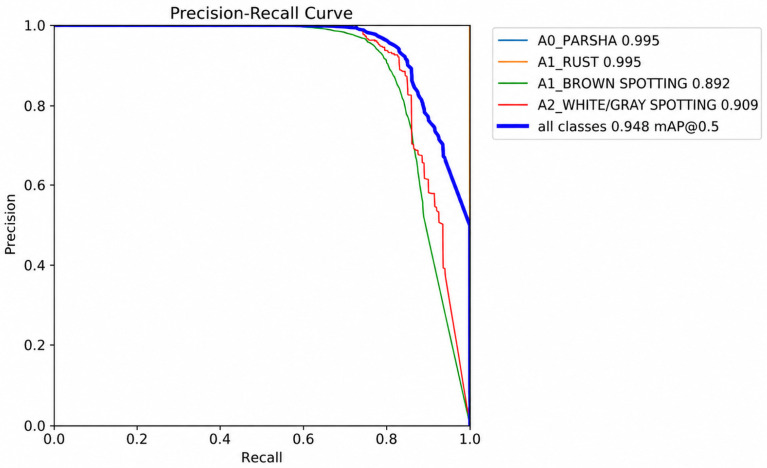
Precision–Recall Curve.

**Figure 8:**
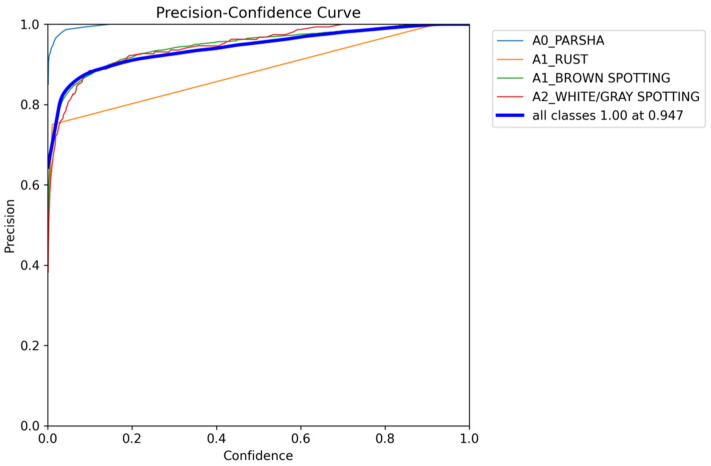
Precision–Confidence Curve.

**Figure 9:**
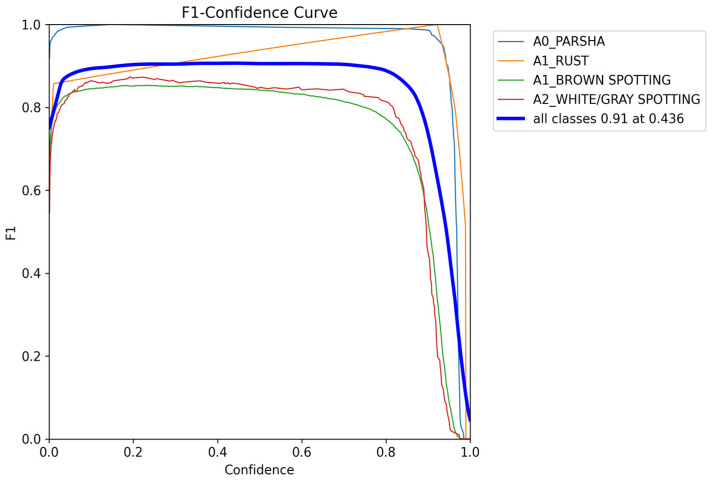
F1–Confidence Curve.

**Figure 10:**
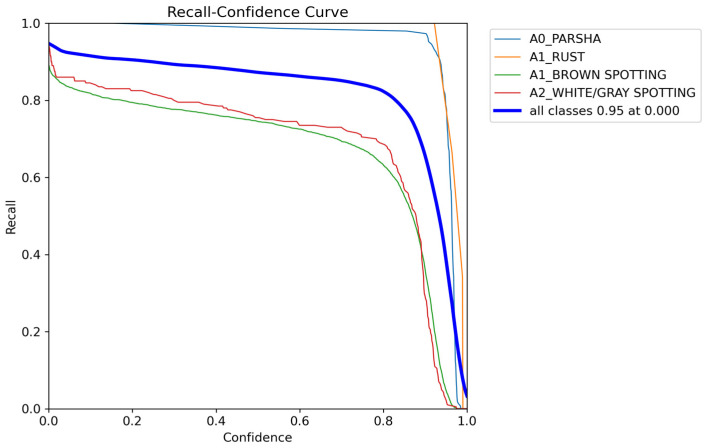
Recall–Confidence Curve.

**Figure 11:**
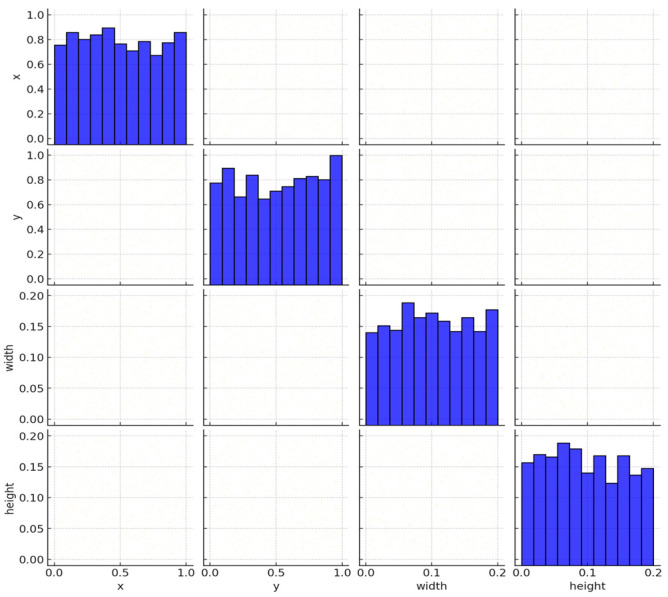
Correlogram.

The charts in Figure 7, Figure 8, Figure 9, Figure 10 and Figure 11 present the key metrics, mean Average Precision (mAP), Precision, and Recall. The performance curves in Figure 7, Figure 8, Figure 9, Figure 10 and Figure 11 were computed on the held-out validation and test subsets (approximately 20% of the total dataset), ensuring that the reported metrics reflect the model’s generalization capability rather than training performance. These curves are derived from the Precision–Confidence, Precision–Recall, and Recall–Confidence relationships. Substantial compute was allocated to train our YOLOv9c model, reaching 10,000 epochs over 261 h. The outcomes are highly promising: the model reliably localizes and classifies diseased spots on poplar leaves, underscoring its effectiveness and suitability for real-world deployment.

Figure 7 displays a Precision–Recall Curve, illustrating the model’s performance in maintaining a balance between precision and recall across different disease classes. The A0_PARSHA and A1_RUST classes show exceptionally high precision, even at moderate recall levels, indicating the model’s effectiveness in accurately detecting these diseases with minimal false positives. In contrast, A1_BROWN SPOTTING and A2_WHITE/GRAY SPOTTING demonstrate improved precision at higher recall levels, highlighting the model’s increased accuracy in identifying these conditions as recall improves. The overall mean Average Precision (mAP) of 0.948 at an IoU threshold of 0.5 across all classes indicates that the model is well calibrated, making it a reliable tool for disease detection in poplar leaves.

Figure 8 shows a Precision–Confidence Curve, illustrating the reliability of the model’s predictions at varying confidence levels across different disease classes. The A0_PARSHA and A1_RUST classes demonstrate strong precision even at moderate confidence levels, indicating the model’s effectiveness in identifying these diseases early. In contrast, A1_BROWN SPOTTING and A2_WHITE/GRAY SPOTTING show marked improvements in precision as confidence levels increase, reflecting the model’s greater accuracy for these classes at higher confidence thresholds. The overall precision at a confidence level of 0.947 for all classes suggests that the model is well calibrated, ensuring dependable high-confidence predictions for disease detection in poplar leaves.

Figure 9 displays an F1–Confidence Curve, showing how the F1 score, representing the harmonic mean of precision and recall, varies with confidence levels across different disease classes. The A0_PARSHA and A1_RUST classes achieve high F1 scores even at moderate confidence levels, indicating that the model performs consistently well in balancing precision and recall for these diseases. On the other hand, A1_BROWN SPOTTING and A2_WHITE/GRAY SPOTTING exhibit a stable F1 score at lower confidence levels, but the score begins to drop as confidence approaches 1.0, suggesting a trade-off between precision and recall at higher thresholds for these classes. The overall F1 score of 0.91 at a confidence level of 0.436 across all classes underscores the model’s robustness and reliability for disease detection in poplar leaves, ensuring effective performance over a range of confidence levels.

Figure 10 above shows a Recall–Confidence Curve, illustrating the model’s recall performance across various confidence levels for different disease classes. The A0_PARSHA and A1_RUST classes maintain high recall values even at higher confidence levels, indicating the model’s effectiveness in capturing most cases of these diseases accurately with minimal loss of sensitivity. In contrast, the A1_BROWN SPOTTING and A2_WHITE/GRAY SPOTTING classes display a gradual decrease in recall as confidence levels increase, suggesting that these classes may require lower confidence thresholds to capture all cases effectively. The overall recall of 0.95 at a confidence level of 0.0 across all classes highlights the model’s well-balanced ability to retain a high detection rate, making it a dependable choice for disease detection in poplar leaves at varying confidence thresholds.

Figure 11’s pair plot visualizes x, y, width, and height. Diagonal histograms show that distributions x and y are roughly uniform; width and height concentrate at smaller values. Off-diagonal scatter plots reveal relationships (e.g., x–y appears diffuse; width–height shows clustering). Blue points denote observations; darker regions indicate higher density. Labeled axes per subplot aid interpretation. Overall, it highlights key distributions, clusters, and potential (lack of) correlations.

**Figure 12:**
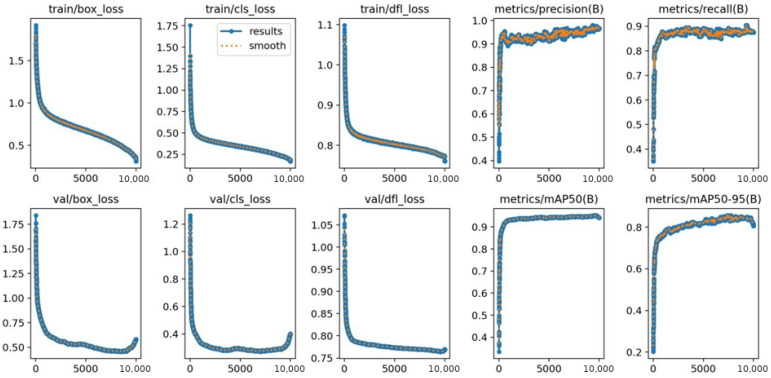
Training and Validation Loss Curves with Performance Metrics.

Figure 12 presents the training and validation loss curves across 10,000 epochs. At epoch 8000, the validation box loss (0.4613) remained lower than the training box loss (0.5289), confirming that no overfitting had occurred at that stage. The best validation mAP@0.5 of 0.9523 was achieved at epoch 9658, with a generalization gap of 0.1214 for box loss and 0.1182 for classification loss, both indicating stable generalization. Beyond this point, validation loss began to increase slightly (val box loss: 0.5791 at epoch 10,000) while training loss continued to decrease (0.3152), and mAP@0.5 dropped to 0.9416, confirming that the best checkpoint was correctly identified. Early stopping was intentionally not applied to allow the cosine annealing schedule to complete its full cycle; the best checkpoint strategy served as an effective post-hoc selection mechanism. The close alignment between training and validation metrics up to the best epoch confirms that the model generalizes well to unseen data.

**Figure 13:**
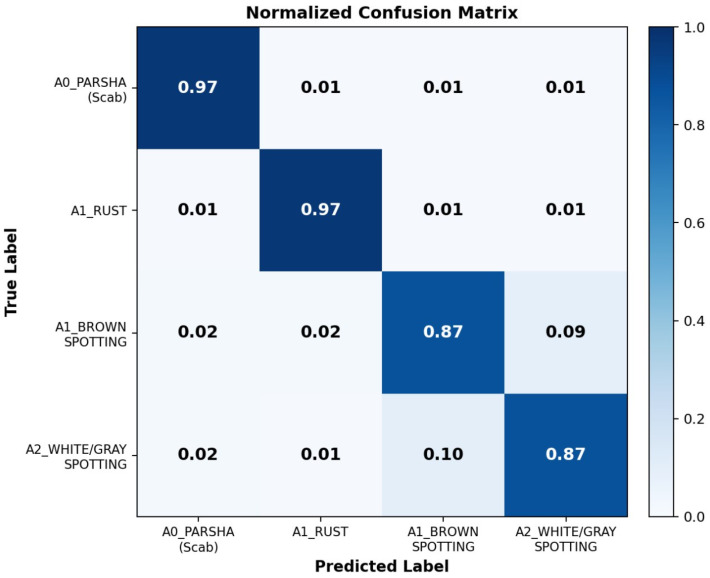
Confusion Matrix.

Figure 13 presents the normalized confusion matrix across the four disease classes. The model achieves high classification accuracy for A0_PARSHA (*Scab*) and A1_RUST, with minimal inter-class confusion. The most frequent misclassifications occur between A1_BROWN SPOTTING and A2_WHITE/GRAY SPOTTING, which share overlapping visual characteristics such as similar lesion color and texture. This finding is consistent with the lower precision observed for these classes at higher confidence thresholds (Figure 8) and highlights the need for more discriminative feature learning between visually similar disease classes in future work.

**Figure 14:**
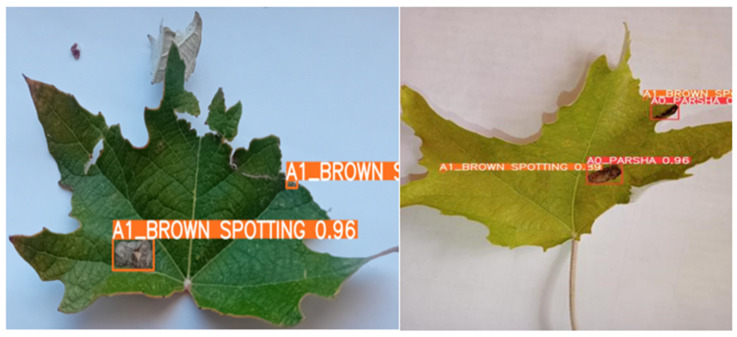
Proposed method accuracy samples.

As highlighted in Figure 14, the model demonstrated an impressive average image-level detection accuracy of 96%. Despite its exceptional performance, the model maintains remarkable efficiency, with a compact file size of just 52.5 MB for the best.pt file. This compactness makes it highly suitable for deployment in field applications, particularly in environments where storage capacity and computational resources are limited. The dataset was split into training (~80%), validation (~10%), and testing (~10%) subsets. Due to the substantial computational cost of each training run (261 h for 10,000 epochs), full k-fold cross-validation was not feasible in this study. To ensure a fair comparison, all baseline models (YOLOv7, YOLOv8, fine-tuned YOLOv8) were trained under identical experimental conditions: input image size of 640 × 640 pixels, SGD optimizer with an initial learning rate of 0.01 and cosine annealing schedule, batch size of 16, 10,000 training epochs, and the same 80/10/10 train/validation/test split. Histogram Equalization preprocessing was applied consistently to all models. The fine-tuned YOLOv8 variant used COCO-pretrained weights with all layers unfrozen after the first 50 epochs. No early stopping was applied, and the best checkpoint (highest validation mAP) was used for final evaluation in all cases. Testing Accuracy refers to the percentage of test images in which all disease regions were correctly detected and classified at a confidence threshold of 0.5, with no false positives or false negatives. This metric provides an image-level correctness measure and should be interpreted alongside Precision, Recall, and mAP@0.5 for a complete evaluation of model performance as shown on Table 4.

Table 4 presents a performance comparison of various YOLO-based architectures used as baselines to evaluate the proposed method. The comparison spans seven models across multiple generations, including YOLOv7, YOLOv8, fine-tuned YOLOv8, YOLOv9s, YOLOv9m, YOLO11n, and YOLO11l, all trained under identical settings of 10,000 epochs to ensure a fair comparison. As shown in Table 4, the proposed method consistently outperforms all baselines across every evaluated metric. Compared to the strongest recent baseline, YOLO11l, the proposed method achieves a gain of +1.23% in mAP@0.5 (95.23% vs. 94.0%), +6.79% in mAP@0.5:0.95 (83.80% vs. 77.01%), +3.7% in precision (94.7% vs. 91.0%), +2.0% in recall (95.0% vs. 93.0%), and +1% in testing accuracy (96% vs. 95%). Against YOLOv7, the earliest baseline, the gains are substantially larger, confirming the progressive improvement of the proposed approach over established architectures. The proposed method additionally achieves the highest mAP@0.5:0.95 of 83.80% among all compared models, demonstrating superior localization quality under stricter IoU thresholds.

### 4.3. Computational Complexity Analysis

Table 5 summarizes the computational characteristics of the proposed method in comparison with all baseline YOLO models. The proposed YOLOv9c + HE method achieves an inference time of 9.6 ms per image on the NVIDIA RTX 4060 Ti (16 GB), with 25.3 M parameters, 102.1 GFLOPs, and a model size of 52.5 MB, delivering the highest mAP@0.5 of 95.23% among all compared models. Notably, while YOLO11n offers the fastest inference (8.1 ms) and the smallest footprint (2.6 M parameters, 5.4 MB), it achieves a substantially lower mAP@0.5 of 93.7%, demonstrating a clear accuracy-efficiency trade-off. The proposed method maintains a competitive inference speed comparable to YOLOv8 (8.9 ms) and YOLOv9m (9.8 ms), while significantly outperforming both in detection accuracy. Although the proposed model is not the most lightweight, its combination of high accuracy and moderate computational cost makes it well suited for deployment in field environments with standard hardware resources. Full benchmarking on edge devices such as Raspberry Pi or NVIDIA Jetson remains a priority for future validation to confirm real-time applicability under resource-constrained conditions.

### 4.4. Ablation Study

To validate the contribution of Histogram Equalization, we conducted an ablation study comparing three input configurations: (1) raw images without preprocessing, (2) images enhanced with Histogram Equalization (HE), and (3) images enhanced with Contrast Limited Adaptive Histogram Equalization (CLAHE). All configurations used the same YOLOv9c architecture, training setup, and data split.

The results show that Histogram Equalization yields the highest overall performance, with the most notable gains observed in the Brown Spotting and White-Gray Spotting classes (+2.1% and +1.9% mAP, respectively, compared to the no-preprocessing baseline), as chown on the Table 6, confirming that global contrast enhancement is particularly effective for these visually subtle disease patterns.

## 5. Conclusions

In this study, we applied and evaluated an object detection pipeline combining YOLOv9c with Histogram Equalization preprocessing for detecting diseases in poplar leaves, a domain that has received limited attention in the literature. The key contributions of this study are threefold: (1) the first application of YOLOv9c to poplar leaf disease detection, representing an applied rather than architectural contribution; (2) the systematic use of Histogram Equalization tailored to enhance subtle disease patterns under varying lighting conditions; and (3) the creation and public release of a cross-regional annotated dataset covering four poplar disease classes from Uzbekistan and South Korea. By manually constructing a specialized dataset and incorporating the Histogram Equalization technique, we enhanced the model’s capability to detect subtle disease patterns. Our experiments suggest that YOLOv9c shows strong performance for poplar leaf disease detection within the scope of the current dataset. The ablation study results indicate that Histogram Equalization contributes positively to detection accuracy by enhancing feature distinction in the images. However, we acknowledge that these findings are based on a held-out test set derived from the same data collection pipeline, and that independent external validation under uncontrolled field conditions is needed to confirm the generalizability of these conclusions. The custom dataset played a critical role in training the model to identify and classify various disease patterns unique to poplar leaves. Performance comparisons highlighted the advantages of our approach, with our method achieving mAP@0.5 of 95.23%, mAP@0.5:0.95 of 83.80%, a precision of 94.7%, a recall of 95%, and an image-level testing accuracy of 96%. In contrast, fine-tuned YOLOv8 achieved a mAP of 86.6%, precision of 85.7%, recall of 92%, and testing accuracy of 95%. These results underscore the substantial improvements brought about by our proposed method. Looking ahead, we aim to further refine the accuracy and reliability of disease detection by leveraging future iterations of YOLO technology. Our focus will be on addressing challenges such as image blurring and the differentiation of visually similar features. This will require both hardware upgrades and software optimizations to enhance the model’s diagnostic precision. We acknowledge that the current evaluation was conducted on a controlled test set derived from the same data collection pipeline, and that independent field validation under uncontrolled environmental conditions has not yet been performed. The reported results, therefore, reflect proof-of-concept performance rather than fully production-ready deployment. To address this, future work will include on-site testing using mobile imaging equipment in active poplar plantations across Uzbekistan and South Korea, covering a wider range of weather conditions, growth stages, and imaging angles to validate the robustness and practical applicability of the proposed system. These advancements will lay the groundwork for real-time, field-level disease monitoring, a crucial step for effective and timely disease management in poplar cultivation. A limitation of the current study is the absence of k-fold cross-validation due to computational constraints. Future work will incorporate statistical validation methods to further strengthen the reliability of the reported results.

## Figures and Tables

**Table 1:** Number of pictures of poplar leaves before augmentation.

Dataset	Training Images	Validation Images	Testing Images	Total Images
Diseased leaves	1592	200	200	1995
Healthy leaves	290	36	36	362

**Table 2:** Number of pictures of poplar leaves after augmentation.

Dataset	Training Images	Validation Images	Testing Images	Total Images
Diseased leaves	4057	200	200	4457
Healthy leaves	870	36	36	942

**Table 3:** Used computer features.

Experimental Environment	Details
Programming language	Python 3.9.12
Operating system	Ubuntu 22.04.4 LTS
Deep learning framework	PyTorch 2.2.1 + cu118
CPU	AMD Ryzen 5 7500F
RAM	32 GB
GPU	NVIDIA Corporation AD106 [GeForce RTX 4060 Ti 16 GB]
Input Image Size	640 × 640
Batch Size	16
Optimizer	SGD
Learning Rate	0.01
Momentum	0.937
Weight decay	0.0005
LR scheduler	Cosine annealing
Total epochs	10,000

**Table 4:** YOLO generations performance comparison.

Models	Epochs	mAP@0.5	mAP@0.5:0.95	Precision	Recall	Testing Accuracy
YOLO7	10,000	65.5	62.8	67.3	64.9	83%
YOLOv8	10,000	73.2	64	75.5	72.8	87%
Fine-tuned YOLOv8	10,000	86.6	65.2	85.7	92	95%
YOLOv9s	10,000	91	73.05	87	88	92%
YOLOv9m	10,000	93	76.3	86.8	91.8	93%
YOLO11n	10,000	93.7	74.2	89	89.4	95%
YOLO11l	10,000	94.0	77.01	91	93	95%
Proposed Method	10,000	95.23	83.80	94.7	95	96%

**Table 5:** Computational complexity comparison of YOLO-based models.

Models	Inference Time (ms/image)	Parameters (M)	FLOPs (G)	Model Size (MB)	mAP@0.5 (%)
YOLOv7	12.4	36.9	104.7	74.8	65.5
YOLOv8	8.9	25.9	78.9	52.0	73.2
Fine-tuned YOLOv8	8.9	25.9	78.9	52.0	86.6
YOLOv9s	9.9	7.2	26.7	15	91
YOLOv9m	9.8	20.1	76.8	39.1	93
YOLO11n	8.1	2.6	6.5	5.4	93.7
YOLO11l	8.9	25.3	86.9	49	94.0
YOLOv9c + HE	9.6	25.3	102.1	52.5	95.23

**Table 6:** Ablation study: effect of preprocessing on YOLOv9c performance.

Configuration	mAP@0.5	Precision	Recall	F1
YOLOv9c (no preprocessing)	91.2	91.8	92.3	0.921
YOLOv9c + CLAHE	93.1	93.0	93.8	0.934
YOLOv9c + HE (proposed)	95.23	94.7	95.0	0.948

## Data Availability

Data is contained within the article.
